# Ginsenoside compound-K attenuates OVX-induced osteoporosis via the suppression of RANKL-induced osteoclastogenesis and oxidative stress

**DOI:** 10.1007/s13659-023-00405-z

**Published:** 2023-11-09

**Authors:** Lingli Ding, Zhao Gao, Siluo Wu, Chen Chen, Yamei Liu, Min Wang, Yage Zhang, Ling Li, Hong Zou, Guoping Zhao, Shengnan Qin, Liangliang Xu

**Affiliations:** 1 Key Laboratory of Orthopaedics and Traumatology, Lingnan Medical Research Center, The First Affiliated Hospital of Guangzhou University of Chinese Medicine, Guangzhou University of Chinese Medicine, Guangzhou, China; 2 Er Sha Sports Training Center of Guangdong Province, Guangzhou, China; 3 School of Basic Medical Science, Guangzhou University of Chinese Medicine, Guangzhou, China; 4 Engineering Laboratory for Nutrition, Shanghai Institute of Nutrition and Health, Chinese Academy of Sciences, Shanghai, China; 5 Master Lab for Innovative Application of Nature Products, National Center of Technology Innovation for Synthetic Biology, Tianjin Institute of Industrial Biotechnology, Chinese Academy of Sciences, Tianjin, China; 6 CAS Key Laboratory of Quantitative Engineering Biology, Shenzhen Institute of Synthetic Biology, Shenzhen Institute of Advanced Technology, Chinese Academy of Sciences, Shenzhen, China; 7 CAS-Key Laboratory of Synthetic Biology, CAS Center for Excellence in Molecular Plant Sciences, Shanghai Institute of Plant Physiology and Ecology, Chinese Academy of Sciences, Shanghai, China; 8 Bio-Med Big Data Center, Shanghai Institute of Nutrition and Health, Chinese Academy of Sciences, Shanghai, China; 9 State Key Laboratory of Genetic Engineering, Department of Microbiology and Immunology, School of Life Sciences, Fudan University, Shanghai, China; 10 Department of Microbiology, The Chinese University of Hong Kong, Hong Kong, China; 11 Department of Orthopaedics, Guangzhou Institute of Traumatic Surgery, Guangzhou Red Cross Hospital, Medical College, Jinan University, Guangzhou, China

**Keywords:** Ginsenoside CK, NF-κB signaling pathway, Osteoporosis, Oxidative stress

## Abstract

Osteoporosis (OP), a systemic and chronic bone disease, is distinguished by low bone mass and destruction of bone microarchitecture. Ginsenoside Compound-K (CK), one of the metabolites of ginsenoside Rb1, has anti-aging, anti-inflammatory, anti-cancer, and hypolipidemic activities. We have demonstrated CK could promote osteogenesis and fracture healing in our previous study. However, the contribution of CK to osteoporosis has not been examined. In the present study, we investigated the effect of CK on osteoclastogenesis and ovariectomy (OVX)-induced osteoporosis. The results showed that CK inhibited receptor activator for nuclear factor-κB ligand (RANKL)-mediated osteoclast differentiation and reactive oxygen species (ROS) activity by inhibiting the phosphorylation of NF-κB p65 and oxidative stress in RAW264.7 cells. In addition, we also demonstrated that CK could inhibit bone resorption using bone marrow-derived macrophages. Furthermore, we demonstrated that CK attenuated bone loss by suppressing the activity of osteoclast and alleviating oxidative stress in vivo. Taken together, these results showed CK could inhibit osteoclastogenesis and prevent OVX-induced bone loss by inhibiting NF-κB signaling pathway.

**Graphical Abstract:**

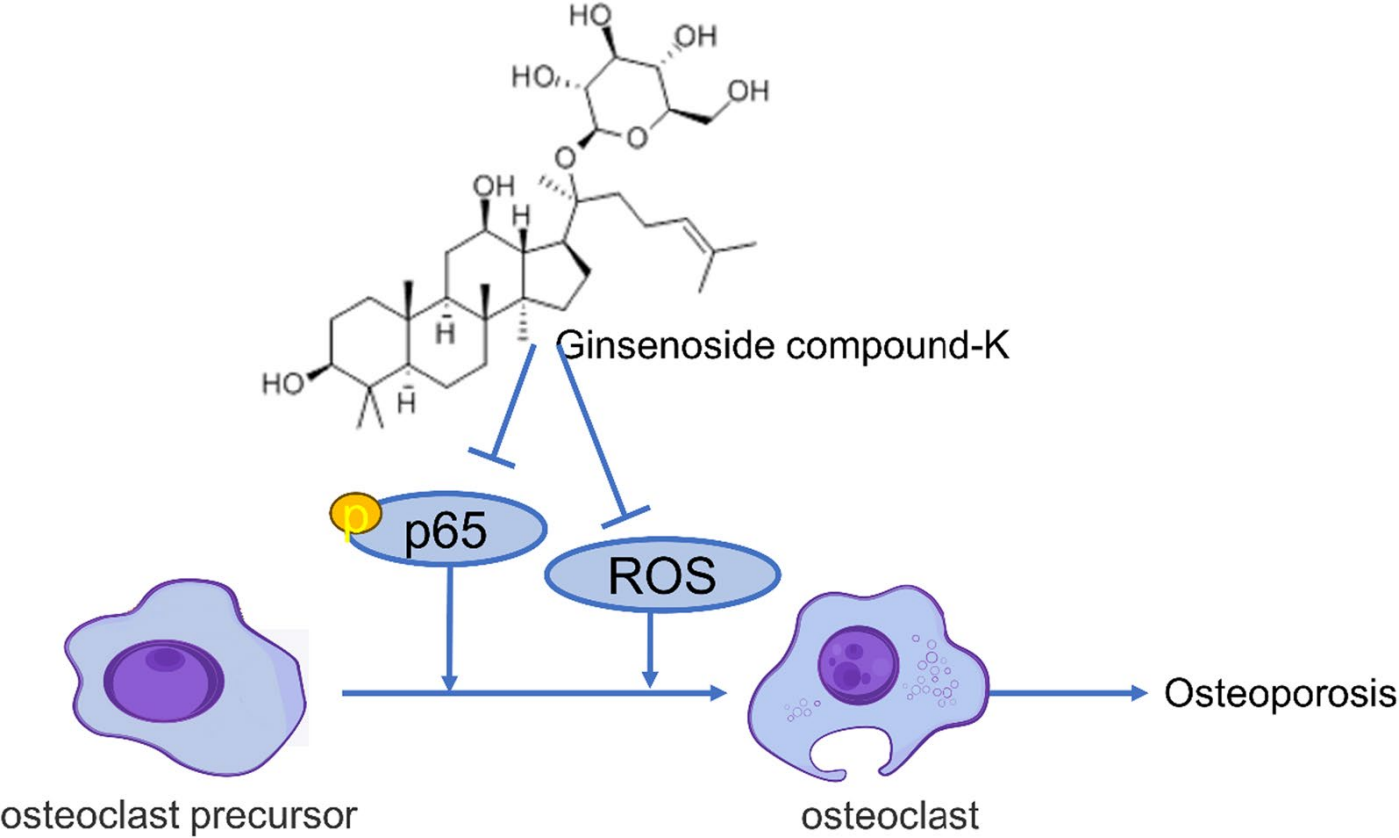

**Supplementary Information:**

The online version contains supplementary material available at 10.1007/s13659-023-00405-z.

## Introduction

Osteoporosis (OP) is a serious clinical problem that usually occurs after menopause with accelerated bone loss. It raises the risk of a fragility fracture, which causes an enormous burden on society. With the aging of society, OP is becoming more widespread. As population demographics change, the global incidence of hip fractures is predicted to hit 6 million by 2050. OP happens when the speed of bone resorption by osteoclasts exceeds the speed of bone formation by osteoblasts, resulting in reduced bone density [1]. Osteoclasts are exclusively bone-resorbing cells, playing a crucial role in bone homeostasis. Receptor activator for nuclear factor-κB ligand (RANKL) stimulates osteoclastogenesis, which triggers bone loss during postmenopausal osteoporosis [2]. Several drugs for the prevention and therapy of OP are currently in use for postmenopausal females, such as bisphosphonates and calcitonin. However, there are published reports of the side effects of these drugs in clinical situations. Therefore, it is necessary to explore better treatments for postmenopausal osteoporosis to reduce fractures and improve the life quality of patients.

Oxidative stress is recognized as an essential causative factor in osteoporosis [3]. Reactive oxygen species (ROS), such as hydrogen peroxide (H_2_O_2_), lipid peroxides, hydroxyl, superoxide radicals, and singlet oxygen are produced as major by-products in oxygen-demanding biological cells, inducing oxidative stress [4]. The formation and activation of osteoblasts and osteoclasts are all tightly associated with oxidative stress. For example, H_2_O_2_ has been shown to inhibit osteoblastic differentiation and the mRNA level of osteogenesis-associated markers such as osteopontin (OPN), osteocalcin (OCN) and runt-related transcription factor 2 (Runx2) [5]. H2O2 also participates in the promotion of osteoclastogenesis, which could be inhibited by anti-oxidants [6]. In addition, aging is associated with the accumulation of ROS, which is also responsible for bone loss.

Ginseng, a member of the Araliaceae family, is used world widely as a medicinal and edible herb. Ginsenosides are the main active ingredients of ginseng. Ginsenoside Compound-K (CK) has been studied for its anti-inflammatory, hypolipidemic, anti-cancer, and anti-aging activities [7–10]. It has been shown that CK might be a potential drug for rheumatoid arthritis [11]. A scaffold incorporated CK has been shown to be biocompatible and support cell proliferation [12]. In a study of the arthroprotective effect of CK, CK was found to inhibit osteoclastic differentiation in human CD14 + monocytes and RAW264.7 cells [13]. The bioconversion of ginsenoside Rb1 by the fungus Penicillium bainier sp.229 produced compounds that induced osteogenic differentiation, and these compounds contained CK [14]. Our previous study has also proved that CK can promote osteogenic differentiation and accelerate fracture healing [15]. A study predicted the potential of CK for the treatment of osteoporosis based on network pharmacology [16]. However, the effect of CK on osteoporosis has not been well-evidenced and its mechanism of action has not been elucidated. Investigating the effect of CK on osteoclastogenesis and OVX-induced osteoporosis will provide insights into potential therapeutic interventions for postmenopausal osteoporosis.

In this study, we investigated the effect of CK on osteoclast differentiation and its potential mechanisms. Additionally, we evaluated the therapeutic potential of CK for treating ovariectomy-induced osteoporosis.

## Result

### CK suppressed RANKL-induced osteoclastogenesis in vitro

**Fig. 1:**
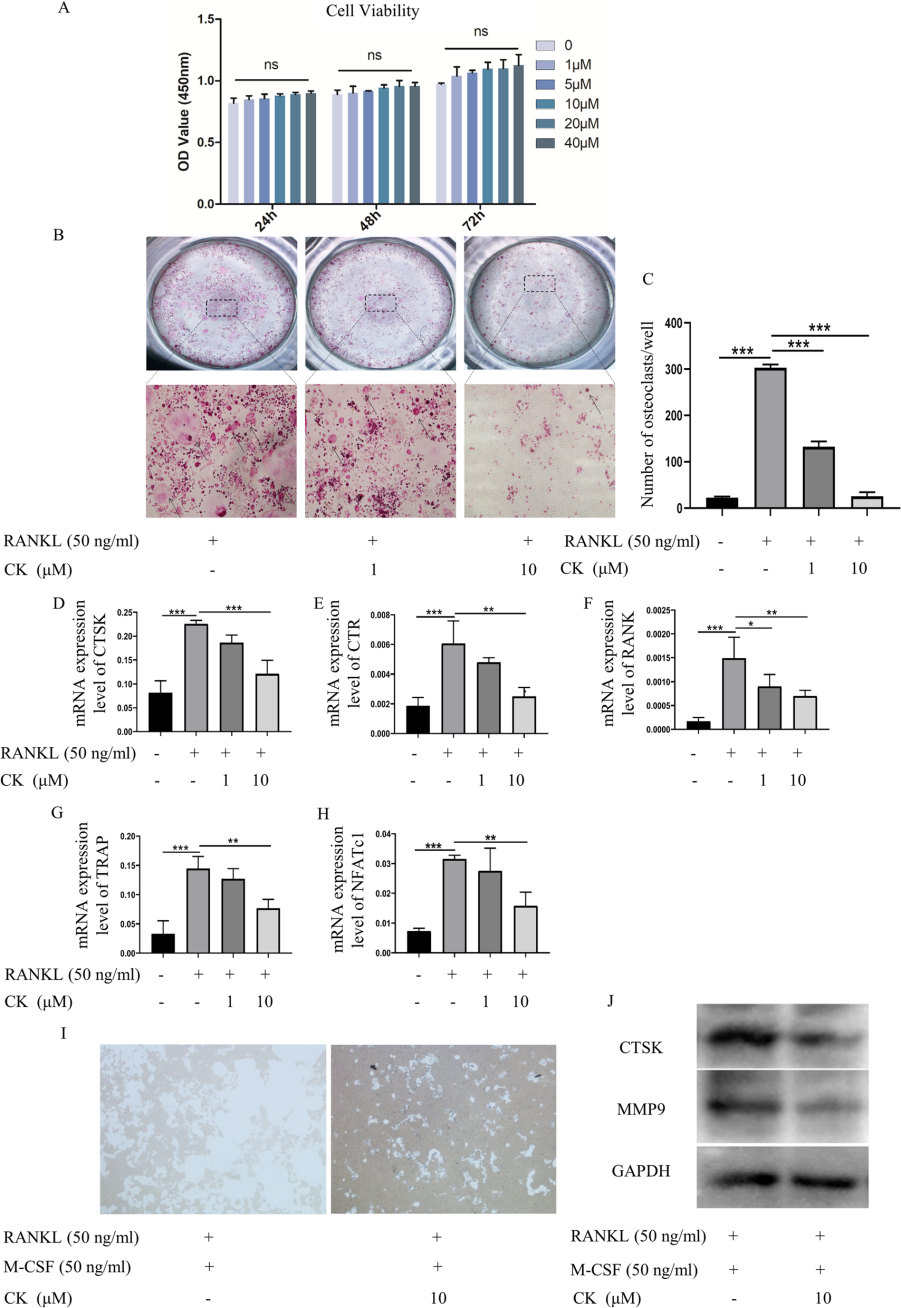
CK inhibited RANKL-induced osteoclastogenesis in vitro. **A** Optical density (OD) values indicated the effects of CK treatment for 24, 48, and 72 h on cell viability of RAW 264.7 cells. n = 5, ns = not statistically significant. **B** Representative images of TRAP staining with indicated concentrations of CK at 5 days after osteoclast differentiation in RAW264.7 cells. **C** Quantification of formed osteoclasts (nuclei > 3), n = 5, ***p < 0.001. **D**–**H** Osteoclastogenesis-related gene expression after 3 days of RANKL-induced osteoclast differentiation without or with CK (1, 10 μM). N = 5, *p < 0.05, **p < 0.01, *** p < 0.001. **I** Representative images of bone resorption without or with CK (10 μM) at 6 days after osteoclast differentiation in bone marrow-derived macrophages (BMMs). **J** Representative western blot images of MMP9 and CTSK at 5 days in RANKL-induced osteoclastogenesis

Apart from our previous study showing CK can promote osteogenic differentiation and angiogenesis, the biochemical targets and molecular mechanism of CK in treating osteoporosis has been predicted through network pharmacology without experimental validation [16]. To further confirm the effect of CK on osteoclastogenesis and osteoporosis, the RAW264.7 cells were treated with various concentrations of CK (0, 1, 5, 10, 20 and 40 μM) for 24, 48, and 72 h (h), and then the Cell Counting Kit-8 (CCK-8) assay showed that CK did not exert a cytotoxic effect on RAW264.7 cells (Fig. 1A). To determine the possible role of CK in osteoclastogenesis, RAW264.7 cells were intervened with 50 ng/mL RANKL at different concentrations of CK (0, 1, 10 μM). After 5 days of induction, tartrate resistant acid phosphatase (TRAP) staining showed that CK inhibited the formation of multinucleated osteoclasts, especially at the CK concentration of 10 μM (Fig. 1B). Quantitative analysis showed that CK significantly reduced the number and surface of RANKL-induced osteoclasts at concentrations of 1 and 10 μM (Fig. 1C). Additionally, reverse transcription-polymerase chain reaction (RT-PCR) was performed at 3 days of induction. The results revealed that CK suppressed osteoclast-specific gene expressions, including, cathepsin K (CTSK), calcitonin receptor (CTR), receptor activator of NF-κB (RANK), TRAP and nuclear factor activated T cell 1 (NFATc1) (Fig. 1D-H)**.** The Bone resorption assay showed that CK (10 μM) visibly inhibited the area of osteoclast resorption (Fig. 1I). Furthermore, western blot showed that CK (10 μM) could inhibit MMP9 and CTSK during osteoclastogenesis (Fig. 1J).

### CK restrained ROS levels by strengthening resistance to oxidative stress

**Fig. 2:**
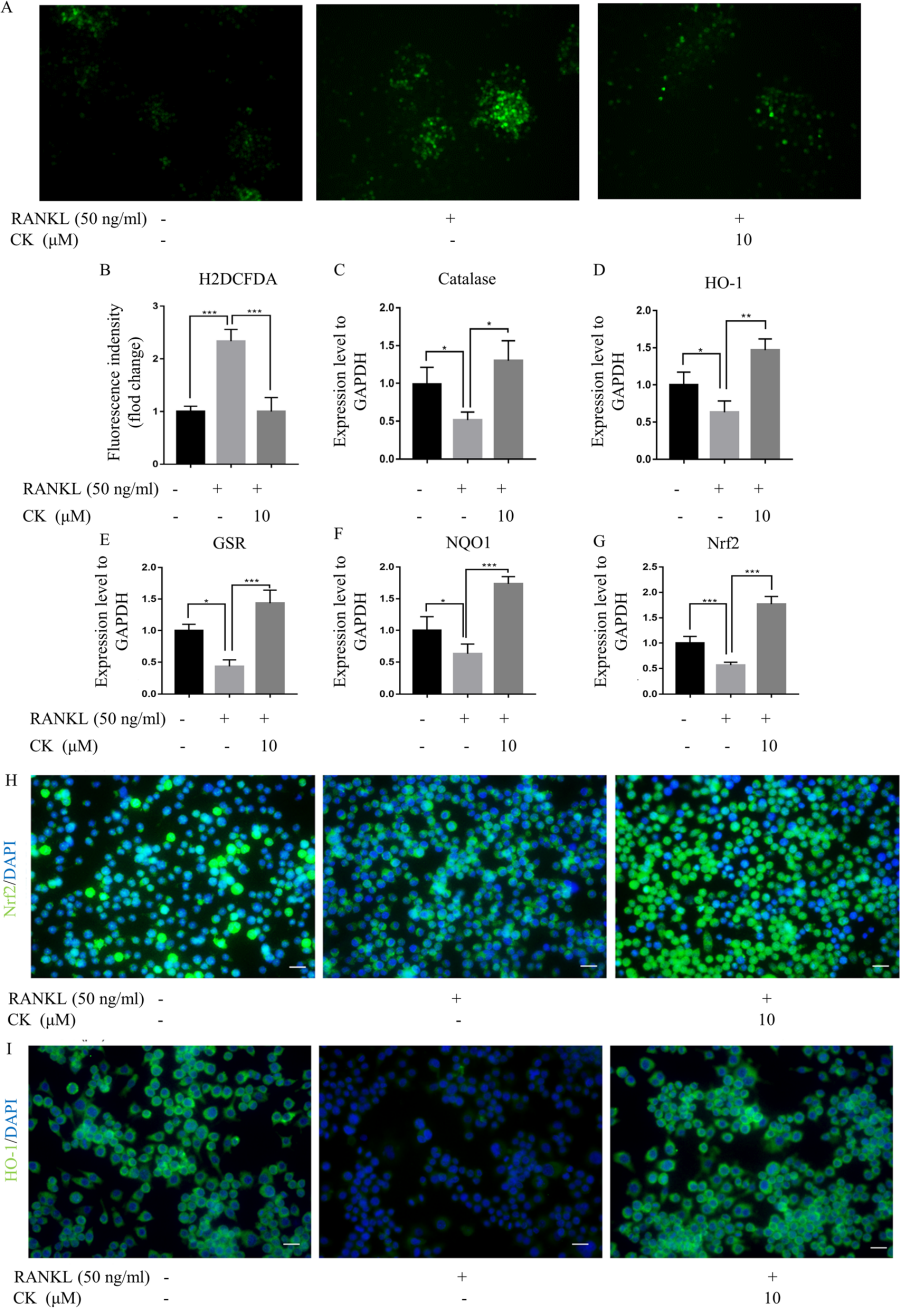
CK restrained ROS levels by strengthening resistance to oxidative stress. **A** ROS level was analyzed by H2DCFDA staining after 3 days of RANKL-induced osteoclast differentiation without or with CK, n = 5, scale bar = 100 μm. **B** Quantification of fluorescence intensity of H2DCFDA staining images, n = 5, ***p < 0.001. **C**–**G** mRNA expressions of catalase, HO-1, GSR, NQO1, and Nrf2 after 3 days of RANKL-induced osteoclast differentiation without or with CK (10 μM). N = 5, *p < 0.05, **p < 0.01, *** p < 0.001. **H** Representative IF images of Nrf2 at 3 days in RANKL-induced osteoclast differentiation, n = 5, scale bar = 20 μm. **I** Representative IF images of HO-1 at 3 days in RANKL-induced osteoclast differentiation, n = 5, scale bar = 20 μm

Next, we carried out 2', 7'-dichlorodihydrofluorescein diacetate (H2DCFDA) immunofluorescence (IF) staining analysis to investigate the effect of CK on ROS levels during RANKL-induced osteoclast formation. The assay was performed after 48 h of RANKL induction and the results showed that CK was able to reduce the intracellular high ROS levels in RANKL-induced osteoclasts (Fig. 2A, B). Meanwhile, we investigated the expression of antioxidant enzymes including catalase, heme oxygenase-1 (HO-1) and glutathione disulfide reductase (GSR), during RANKL-induced osteoclast formation. In RANKL-induced group, GSR, HO-1, and catalase levels were reduced, while they were upregulated after CK treatment (Fig. 2C–E). The expression of the transcription factor for stress response, nuclear factor erythroid 2-related factor 2 (Nrf2), and its target gene NAD(P)H quinone dehydrogenase 1 (NQO1) was also increased by CK treatment of RANKL-induced osteoclasts (Fig. 2F, G). We examined the protein localization of Nrf2 and HO-1 in RAW264.7 cells with the intervention of RANKL and CK. It was found that Nrf2 was exported from the nucleus to the cytoplasm after induction by RANKL, while returned from the cytoplasm to the nucleus by the treatment of CK (Fig. 2H). And in RANKL-induced group, the expression of HO-1 was decreased, while upregulated after CK treatment (Fig. 2I).

### CK suppressed NF-κB signaling pathway

**Fig. 3:**
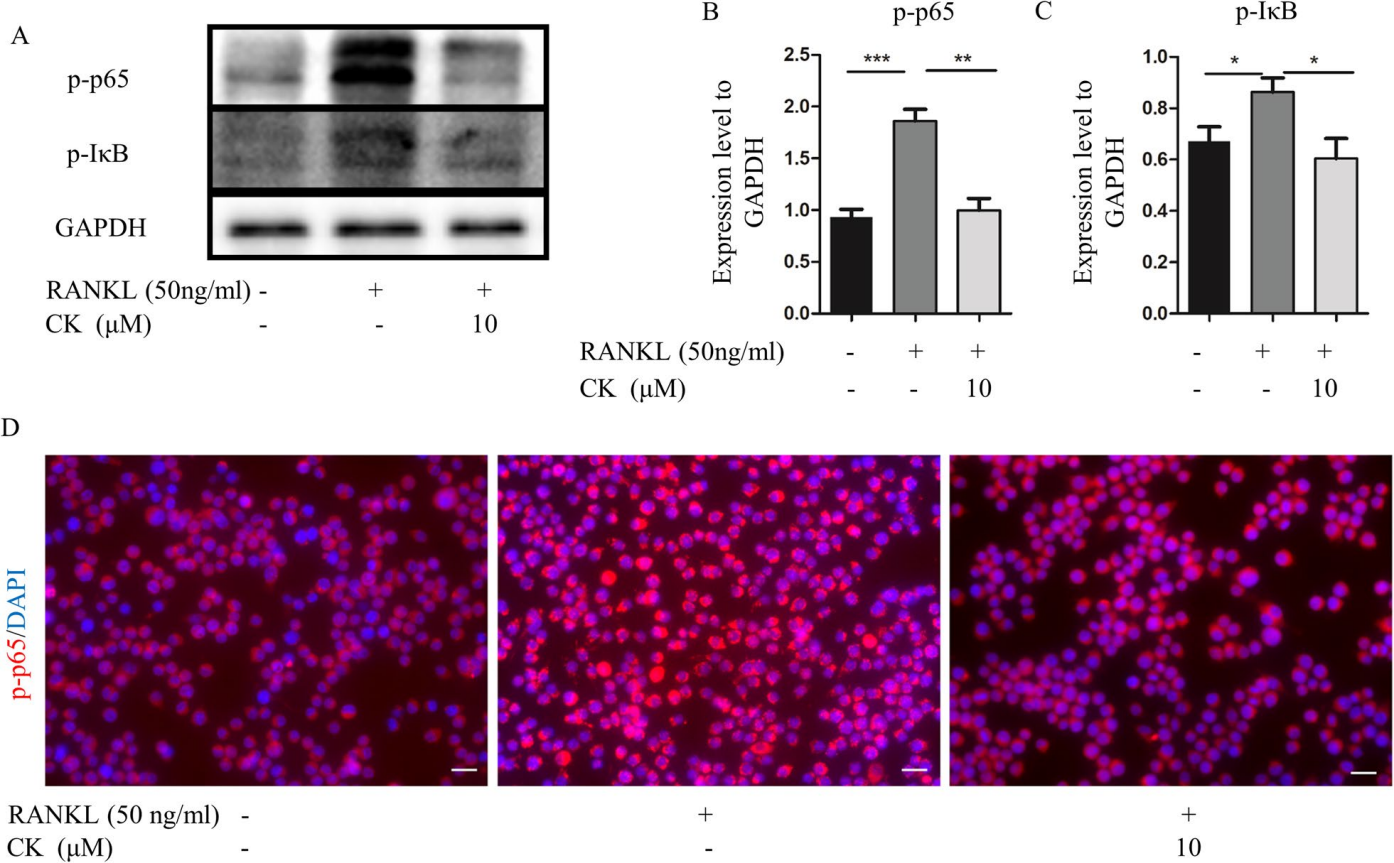
CK suppressed NF-κB signaling pathway. **A**–**C** Representative western blot images and relative quantification of p-p65, and p-IκB at 3 days in RANKL-induced osteoclastogenesis of RAW264.7 cells. n = 5, *p < 0.05, **p < 0.01, *** p < 0.001. **D** Representative IF images of NF-κB p65 at 3 days in RANKL-induced osteoclast differentiation, n = 5, scale bar = 20 μm

NF-κB signaling pathway plays a critical role in osteoclast formation. And the network pharmacology prediction also showed NF-κB signaling pathway might be the target of CK [16]. To confirm the effect of CK on NF-κB pathway stimulated by RANKL, we measured the protein levels of p-IκB and p-p65. The result of western blot showed that CK (10 μM) could inhibit p-IκB, and p-p65 during RANKL-induced osteoclastogenesis (Fig. 3A–C). IF staining showed that the nucleus translocation of p-p65 was suppressed by CK (10 μM) (Fig. 3D). Therefore, our findings suggested that CK (10 μM) suppressed NF-κB signaling pathway to inhibit osteoclast formation.

### CK protected against bone mass loss in ovariectomized mice 

**Fig. 4:**
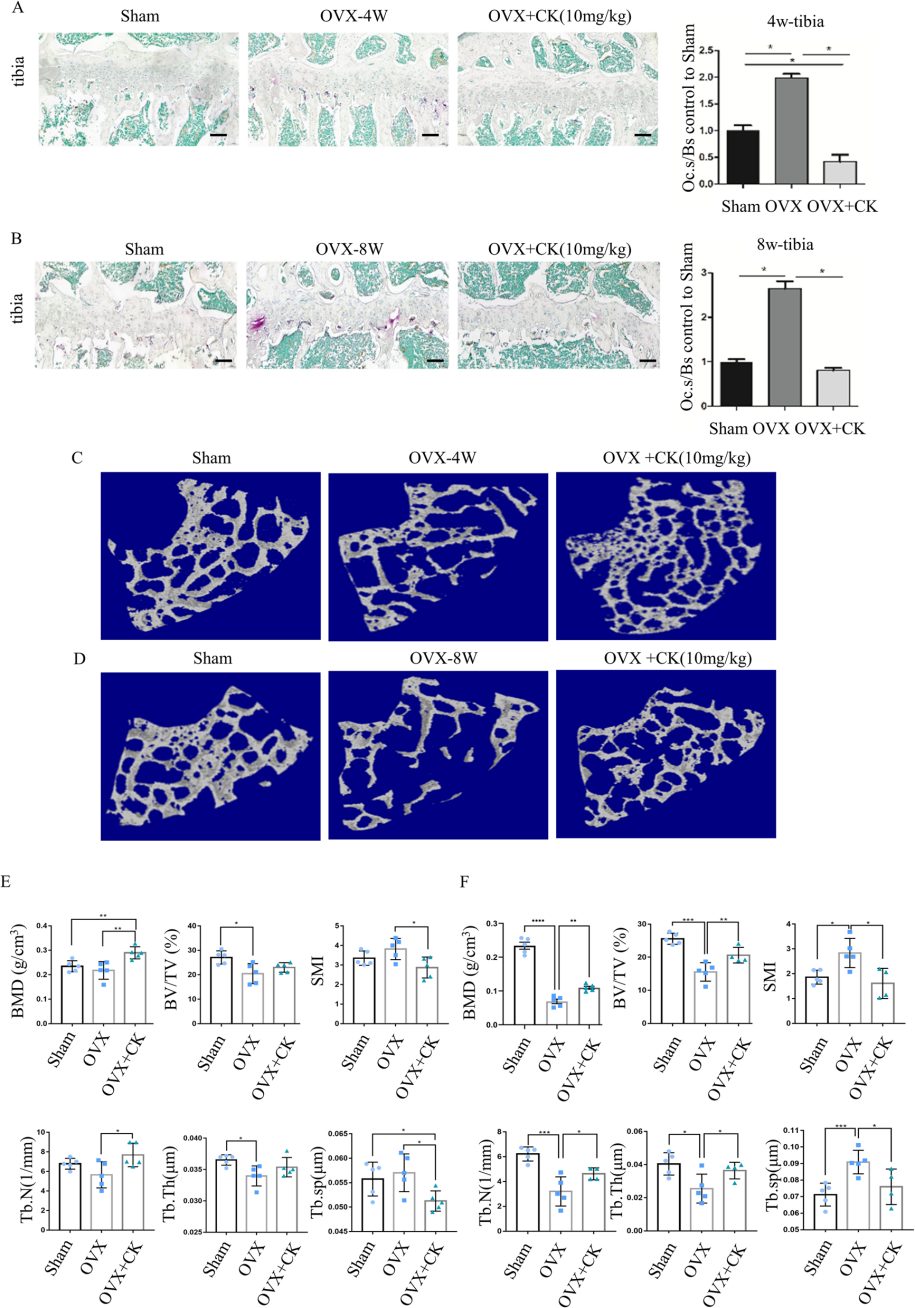
CK protected against bone mass after OVX. **A**, **B** Representative images of TRAP staining and quantification in the sham, OVX, and OVX + CK group mice at 4 weeks (**A**) and 8 weeks (**B**) after ovariectomy, scale bar = 100 μm, n = 5, *p < 0.05. **C**, **D** Representative 3-dimensional micro-CT images of femur bone sections at 4 and 8 weeks after ovariectomy. **E**, **F** Quantitative analysis of bone structural parameters, including BMD, BV/TV, SMI, Tb.Th, Tb.sp, Tb.N in the sham, OVX, and OVX + CK group mice at 4 weeks (**E**) and 8 weeks (**F**) after ovariectomy. n = 5, *p < 0.05

**Fig. 5:**
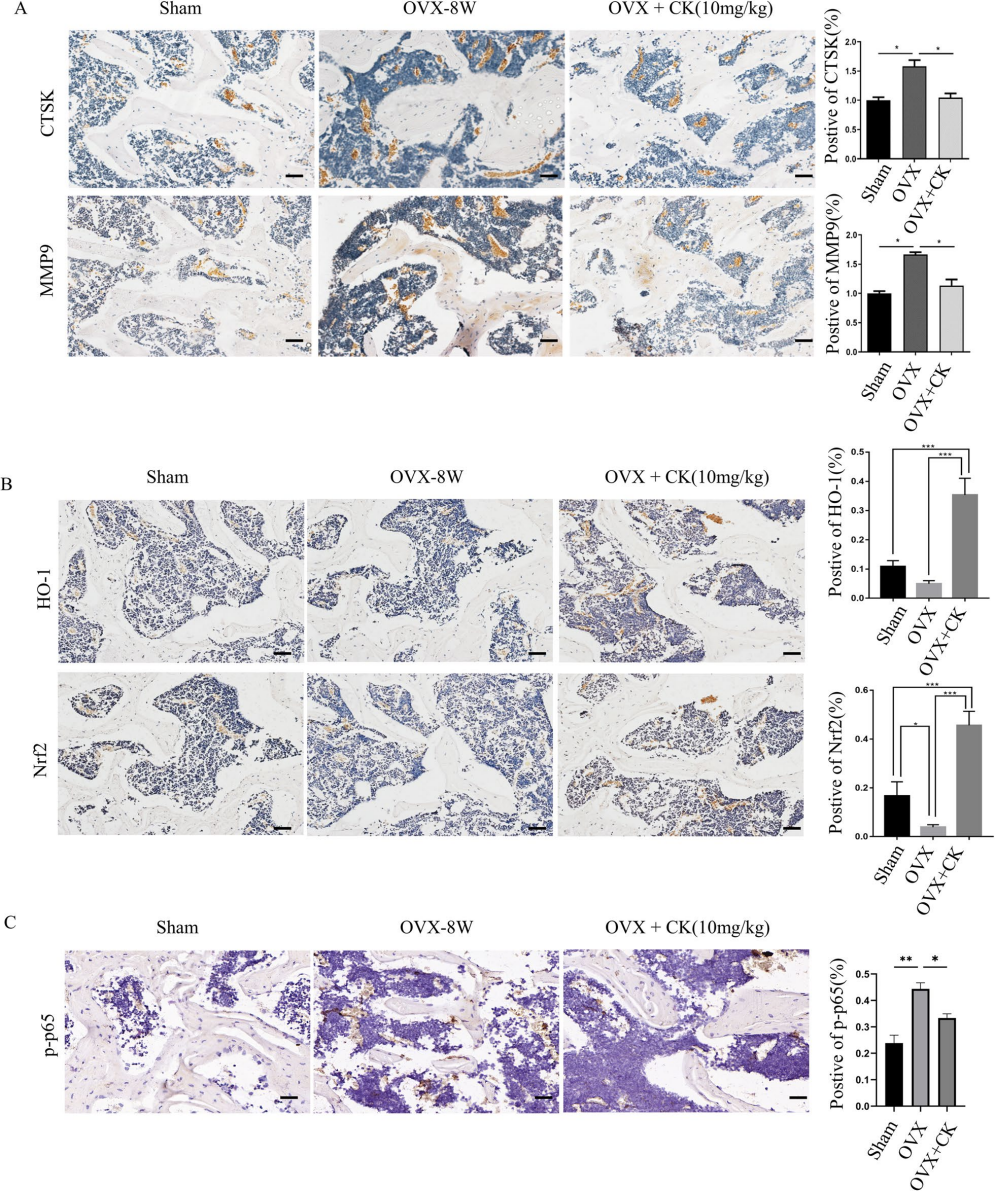
CK inhibited oxidative stress in vivo. **A** Representative IHC images and quantification of MMP9 and CTSK in the sham, OVX, and OVX + CK group mice at 8 weeks after ovariectomy, scale bar = 50 μm, n = 5, *p < 0.05. **B** Representative IHC images and quantification of HO-1 and Nrf2 in the sham, OVX, and OVX + CK group mice at 8 weeks after ovariectomy, scale bar = 50 μm, n = 5, *p < 0.05, **p < 0.01, *** p < 0.001. **C** Representative IHC images and quantification of p-p65 in the sham, OVX, and OVX + CK group mice at 8 weeks after ovariectomy, scale bar = 50 μm, n = 5, *p < 0.05

To confirm the preventive and therapeutic function of CK, CK was intraperitoneally injected into mice after OVX surgery. At 4 and 8 weeks after ovariectomy, the long bones were collected. The TRAP staining demonstrated that CK significantly decreased the number of osteoclasts, and the surface area of the osteoclasts on the bone surface, as compared to those in the OVX group (Fig. 4A, B). Micro-CT analysis showed the bone structure characteristics were significantly improved by CK (10 mg/kg) compared with the OVX group (Fig. 4C–F). Given CK was found to promote osteogenic differentiation in our previous study [14], we examined the effect of CK on the expression of ALP, OPN and OCN (Additional file 1: Fig. S1). The immunohistochemical (IHC) staining results showed that ALP, OPN and OCN were significantly increased in the OVX + CK group, compared to the OVX group. The IHC staining results also indicated that CK treatment decreased the expression of MMP9 and CTSK (Fig. 5A). Since the in vitro results had shown CK could inhibit oxidative stress, so we also detected the expression of HO-1 and Nrf2 in vivo by IHC analysis. The result showed that the protein levels of Nrf2 and HO-1 were significantly decreased in the OVX group, while reversed by CK at 8 weeks after surgery (Fig. 5B). Also, we confirmed the phosphorylated NF-κB p65 was decreased by CK in vivo (Fig. 5C).

## Discussion

Herbal extracts and natural compounds have been widely studied as ideal sources for drug design due to their lesser toxic properties. In our study, we showed that CK had a therapeutic effect on osteoclastogenesis in vitro and inhibited bone loss in vivo, which reveals the potential of CK in OP treatment. Furthermore, in the experiment, we interpreted the potential mechanism of CK affecting bone loss in terms of oxidative stress.

Osteoclasts are the only cells that degrade bone and are key mediators of bone diseases, including osteoporosis [17]. Our experiments showed that CK down-regulated the expression of osteoclast-specific gene levels, such as TRAP, RANK, CTR, CTSK, and NFATc1, and inhibited the formation of osteoclasts, in the meantime, inhibited bone loss, such as osteoclast-specific makers, CTSK and MMP-9. On the other hand, CK promoted bone formation, which is consistent with our previous study [15]. By reducing inflammation, activating Wnt/β-catenin signaling, and promoting the production of bone morphogenetic protein-2 (BMP2), CK has the potential to be an alternative to bone regeneration [12, 14]. Which is consistent with the findings of this experiment as CK increased the expressions of ALP, OPN, and OCN at supplementary materials.

Network pharmacology is a novel research method for discovering new drugs, and it's an excellent way to reflect the interaction between biological molecules and chemical components [18]. Through this method, scholars’ analysis obtained key targets and pathways for CK treatment of osteoporosis, which may reveal the possible mechanism. KEGG pathway analysis uncovered that a majority of the enrichment pathways might be involved [16]. Combined with the KEGG enrichment results, some studies have reported that PI3K-Akt signalling [19], endocrine resistance [20], JAK-STAT signalling [21], reactive oxygen species [22–24], Nrf2/HO-1signaling [22–24], NF-κB signalling [25, 26], HIF-1 signaling [27], FoxO signaling [28], and growth hormone synthesis, secretion and action [29] are associated with bone metabolism and osteoclast differentiation.

Based on the results of network pharmacology, NF-kB, and Nrf2/HO-1 signaling pathways were verified in our study. RANKL signaling is mediated by TRAF6, which leads to the activation of the transcription factor NF-κB, and subsequently activates NFATc1 which is the key osteoclastogenesis regulator [30]. Osteoclast differentiation from mononuclear/macrophage lineages is stimulated by two important cytokines, M-CSF and RANKL [31, 32]. RANKL induces and activates a variety of transcription factors, such as NF-κB, c-FOS, and NFATc1, which are positive regulators of osteoclastogenesis [33]. Our findings suggested that CK reduced NF-κB p-p65 and pIκB, thereby inhibiting bone loss. There is growing evidence that intracellular reactive oxygen species (ROS) accumulation is a key factor in the development of osteoporosis, triggering the formation and function of osteoclasts [34, 35]. Our study showed that CK (10 μM) reduced the intracellular high ROS levels in RANKL-induced osteoclastogenesis. Oxidative stress is recognized as a causal factor in the reduction of bone mineral density in osteoporosis [36]. HO-1 is a downstream target of the Nrf2 and is regarded as a negative regulator of osteoclast differentiation [37]. Our results provided evidence that CK activated the Nrf2/HO-1 signaling pathway to alleviate the oxidative stress and prevent bone loss.

## Conclusions

In conclusion, we demonstrated that CK inhibited RANKL-induced osteoclastogenesis by regulating the NF-kB signaling pathways. In addition, we also found that CK could reduce the intracellular ROS level in vitro, and alleviate oxidative stress in vivo. CK might be a potential chemical for preventing osteoporosis caused by age-related or sex hormone deficiency.

## Materials and methods

### Reagents and antibodies

CK was solubilized in dimethyl sulfoxide (DMSO) and diluted in culture medium or PBS. Fetal bovine serum, Modified Eagle’s Medium of alpha, penicillin, and streptomycin were obtained from PeproTech (New Jersey, USA). Recombinant Murine sRANK Ligand (RANKL) was purchased from R&D (Minnesota, USA). NaOAc3H_2_O, Sodium Tartrate Dihydrate, Ethylene Glycol Monoethyl Ether, Naphthol AS-MX phosphate, and Fast Red Violet LB salt were obtained from Sigma (St. Louis, Missouri, USA). TRIzol reagent and SYBR-Green RT-PCR kit were provided by Takara (Osaka, Japan). Evo M-MLV One Step RT-PCR Kit was supplied by Accurate Biotechnology (Hunan) Co., Ltd, (Changsha, China)CCK-8, RIPA Lysis Buffer, and NcmECL Ultra kit were purchased from NCM Biotech (Suzhou, China). Bone Resorption Assay Kit (CSR-BRA-48KIT) was provided by Cosmo Bio (Tokyo, Japan). Primary antibodies anti-ALP, OPN, GAPDH and OCN were purchased by Santa Cruz Biotechnology (Texas, USA); Primary antibodies anti-MMP9, anti-p-NFκB p65, anti-p-IκB, anti-Nrf2, and anti-HO-1 were purchased from Bioss (Beijing, China); Anti-CTSK was purchased from Sigma (St. Louis, Missouri, USA); and DAPI were obtained from Sigma (St. Louis, Missouri, USA). Secondary antibodies HRP-conjugated Goat Anti-Rabbit IgG, Goat anti-Mouse IgG, Rabbit anti-Goat IgG (H + L) were purchased from Affinity (Jiangsu, China). Mouse Anti-Rabbit IgM/Cy3, Rabbit Anti-Mouse IgM/FITC were supplied from Bioss (Beijing, China). The RAW264.7 cells were obtained fromThermo Scientific (Massachusetts, USA).

### Cytotoxicity assay

The cell viability of RAW 264.7 cells was measured by a CCK-8 kit. RAW 264.7 cells were cultured without or with CK (1, 5, 10, 20, 40 μM) for 24 h, 48 h, and 72 h. And CCK-8 reagent was incubated for 1 h at 37℃. The Optical density (OD) of the samples was detected at 450 nm.

### RNA extraction and RT-PCR

After stimulation with 50 ng/ml RANKL for 3 days in 24-well plates, cells were collected. Total RNA was extracted by TRIzol. Next, Reverse Transcription-Polymerase Chain Reaction (RT-PCR) was performed using Evo M-MLV One Step RT-PCR Kit. The gene expression level was calculated by the 2^–ΔCT^ or 2^–ΔΔCT^ method, and GAPDH was used as the reference for standardization. The primer sequences were shown in Additional file 2: Table S1.

### Bone resorption assay 

Osteoclast activity was determined with the Bone Resorption Assay Kit (Cosmo Bio, CSR-BRA-48KIT), according to the manufacturer's instructions. Briefly, add Fluoresceinamine Labeled Sodium Chondroitin Sulfate (FACS) to each well of a 48-well plate and incubate at 37 °C for 1–2 h. After rinsing with PBS and medium, macrophage bone marrow-derived macrophages (BMMs) are added and incubated. The cultured cells were stimulated with 50 ng/ml RANKL and 50 ng/ml M-CSF to form osteoclasts. With or without CK for 6 days in culture, cells can be removed by treatment with 5% sodium hypochlorite, and after rinsing, photographed with a microscope.

### ROS measurements

Intracellular ROS activity was examined using 2ʹ,7ʹ-dichlorodihydrofluorescein diacetate (H2DCFDA) dye. RAW 264.7 cells were inoculated in 12-well plates. After cell attachment, the medium was exchanged for medium containing RANKL (50 ng/ml), with or without CK (10 μm) for 72 h. Cells were incubated with 5 µM staining solution for 30 min at room temperature avoid light and washed with PBS. H2DCFDA staining images were then taken by a fluorescence microscope (Olympus, IX73L, USA). Fluorescence intensity was measured with Image J software.

### Protein extraction and Western blot analysis

Cells were washed twice with pre-cooling of PBS and then solubilized in RIPA lysis buffer. After freeze–thaw cycling at 4 °C for 0.5 h, the lysate was clarified by centrifugation at 4 °C for 15 min. Total protein from RAW 264.7 cells or BMMs was transferred to PVDF membrane by SDS-PAGE. The PVDF membranes were immersed in a containment solution for 1 h at 37 ℃. The membrane was then incubated with primary antibodies at 4 °C overnight. The primary antibodies included anti-GAPDH, anti-p-IκB, anti-p-p65, anti-MMP9, and anti-CTSK. The membrane was washed and incubated with a secondary antibody for 1 h. The immunoreactions were visualized with BeyoECL Moon.

### In vitro osteoclastogenesis assay

RAW264.7 cells were seeded in 96-well plates at 5 × 10^3^ cells/well. RAW264.7 cells were induced to osteoclasts with RANKL (50 ng/mL), without or with CK (1, 10 μM) for 5 days. After fixing the cells with 10% neutral formalin, the osteoclasts were washed with PBS. Tartrate-resistant acidic phosphatase (TRAP) staining solution 70 μl/well (96-well plate) or 150 μl/well (48-well plate) 10 -30 min at room temp or 37 °C, and wash with H2O and air dry. Multi-nucleated (nuclei ≥ 3) TRAP + cells were counted as osteoclasts (OCs).

### Animal experiments

Eight-week-old Balb/C female mice (19 ± 4.45 g) were obtained from the Center of Experiment Animal of Guangzhou University of Chinese medicine. The study was approved by the Academic Committee on the Ethics of Animal Experiments of the Guangzhou University of Chinese medicine. The mice were randomly divided into the following three groups: Sham (n = 10), OVX + PBS (n = 10), OVX + CK (n = 10, 10 mg/kg). The duration of intraperitoneal injection was 4 and 8 weeks after OVX surgery, respectively. The right tibia and femur were decalcified for histology analysis, while the undecalcified samples on the left were analyzed by bone micro-architecture analysis.

### Micro-CT analysis

The bone trabecular microstructure of the proximal cancellous bone of the right femur was determined using a Skyscan 1176 micro-CT scanner (Bruker micro-CT, Kontich, Belgium). Briefly, the designated scanned femoral region (5 μm/slice, 1 K) was located 1–5 mm from the growth plate-epiphysis junction. Bone mineral density (BMD), trabecular thickness (Tb. Th), trabecular number (Tb. N), bone volume fraction (BV/TV), trabecular separation (Tb. Sp), structural model index (SMI), and femoral 3D images were then obtained from the system software.

### Histology

The tibias and femurs were fixated in 10% buffer formalin overnight, decalcified in 10% EDTA for 21 days, dehydrated, and then paraffin-embedded. Sections were cut into 5 μm thick and the samples were subjected for immunohistochemical (IHC), and TRAP staining. For IHC staining, sections were incubated with 10% BSA at 37 ℃ for 1 h after peroxidase inactivation and antigen retrieval, then incubated with primary antibody overnight at 4 °C and secondary antibody for 1 h at 37 °C. After counterstaining with hematoxylin, image acquisition was performed. The primary antibodies used in this study included anti-MMP9 (1:200), anti-CTSK (1:200), anti-Nrf2 (1:200), anti-HO-1 (1:200) and anti-p-p65(1:100) antibodies. The relative intensity was analyzed by Image J software.

### Immunofluorescence staining

For paraffin section, the slides were dewaxed to distilled water and then performed antigen retrieval. For RAW264.7 cells, cells were seeded in 24-well plates (with coverslips) with 5 × 10^3^ cells /ml and were collected after 48 h without or with 10 μM CK treatment. Fix cells with 4% paraformaldehyde for 15 min. Add 5% BSA to the slides, seal at room temperature for 1 h, and then incubate with primary antibody at 4 °C overnight. Then incubate with fluorescent secondary antibody at 37 °C for 1 h. The primary antibodies used in this study included anti-p-p65 (1:200), and anti-Nrf2 (1:200). DAPI staining was used to visualize the cell nuclei. Images were captured with a fluorescence microscope (Olympus, IX73 L, USA).

### Statistical analysis

Data were analyzed by using GraphPad Prism 7. Quantitative data were represented as mean ± standard deviation (S.D.). Multiple comparisons between groups were statistically analyzed using one-way or two-way analysis of variance (ANOVA). P < 0.05 was regarded as a statistically significant difference.

### Supplementary Information

**Additional file 1: Fig. S1.** Representative IHC images of femur bone sections at 8 weeks after ovariectomy in sham (n = 5), OVX (n = 5), and OVX + CK (n = 5) group mice, scale bar = 50 μm.

**Additional file 2: Table S1.** Primers used for RT-PCR

## Data Availability

The data used and/or analyzed during the current study are available from the corresponding author upon reasonable request.
