# Supplementary material for: Ginsenoside compound-K attenuates OVX-induced osteoporosis via the suppression of RANKL-induced osteoclastogenesis and oxidative stress
Source: Nat Prod Bioprospect. 2023 Nov 9;13(1):49. doi: 10.1007/s13659-023-00405-z (PMC10632357; doi:10.1007/s13659-023-00405-z)
Supplement: Supplementary file 1 — Additional file 1: Fig. S1. Representative IHC images of femur bone sections at 8 weeks after ovariectomy in sham (n = 5), OVX (n = 5), and OVX + CK (n = 5) group mice, scale bar = 50 μm. [file 13659_2023_405_MOESM1_ESM.docx]

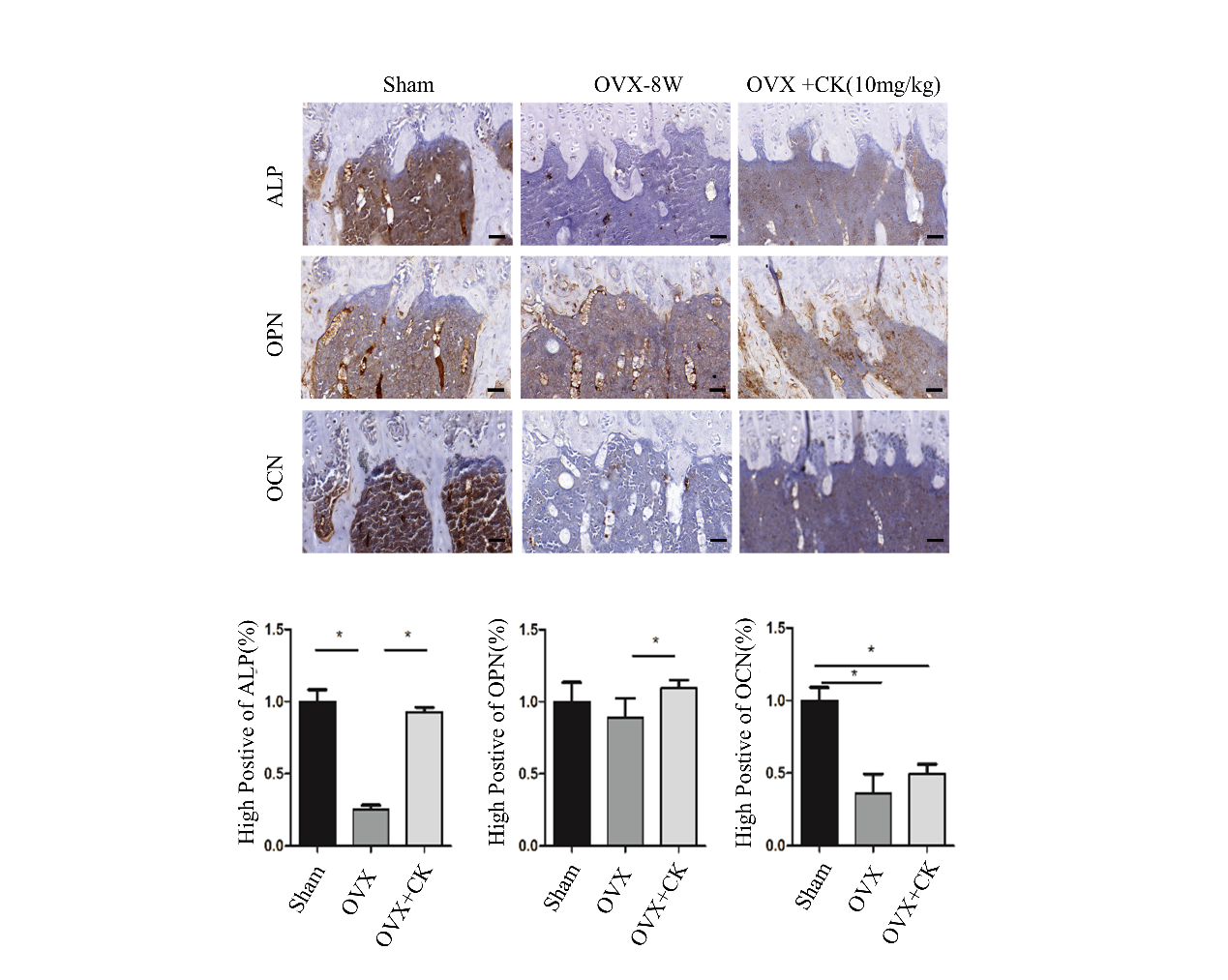


**Supplementary Figure 1.** Representative IHC images of femur bone sections at 8 weeks after ovariectomy in sham (n = 5), OVX (n = 5), and OVX+CK (n = 5) group mice, scale bar = 50μm.
