# Supplementary material for: Ginsenoside compound-K attenuates OVX-induced osteoporosis via the suppression of RANKL-induced osteoclastogenesis and oxidative stress
Source: Nat Prod Bioprospect. 2023 Nov 9;13(1):49. doi: 10.1007/s13659-023-00405-z (PMC10632357; doi:10.1007/s13659-023-00405-z)
Supplement: Supplementary file 2 — Additional file 2: Table S1. Primers used for RT-PCR [file 13659_2023_405_MOESM2_ESM.docx]

Table 1: Primers used for RT-PCR.

| Traget gene | Sequence(5'-3') |
| --- | --- |
| GAPDH | F, AGGTCGGTGTGAACGGATTTG |
|  | R, TGTAGACCATGTAGTTGAGGTCA |
| RANK | F, GGACGGTGTTGCAGCAGAT |
|  | R, GCAGTCTGAGTTCCAGTGGTA |
| CTR | F, GCAACGCTTTCACTTCTGAGA |
|  | R, GTTCCCACTGCATTGTCCACA |
| CTSK | F, GAAGAAGACTCACCAGAAGCAG |
|  | R, TCCAGGTTATGGGCAGAGATT |
| NFATc1 | F, GACCCGGAGTTCGACTTCG |
|  | R, TGACACTAGGGGACACATAACTG |
| TRAP | F, CACTCCCACCCTGAGATTTGT |
|  | R, CATCGTCTGCACGGTTCTG |
| HO-1 | F, AAGCCGAGAATGCTGAGTTCA |
|  | R, GCCGTGTAGATATGGTACAAGGA |
| Nrf2 | F,TCTTGGAGTAAGTCGAGAAGTGT |
|  | R, GTTGAAACTGAGCGAAAAAGGC |
| NQO1 | F, AGGATGGGAGGTACTCGAATC |
|  | R, AGGCGTCCTTCCTTATATGCTA |
| Catalase | F, AGCGACCAGATGAAGCAGTG |
|  | R, TCCGCTCTCTGTCAAAGTGTG |
| GSR | F, GACACCTCTTCCTTCGACTACC |
|  | R, CCCAGCTTGTGACTCTCCAC |
